# Stress “Deafness” Reveals Absence of Lexical Marking of Stress or Tone in the Adult Grammar

**DOI:** 10.1371/journal.pone.0143968

**Published:** 2015-12-07

**Authors:** Hamed Rahmani, Toni Rietveld, Carlos Gussenhoven

**Affiliations:** Department of Linguistics, Radboud University Nijmegen, Nijmegen, The Netherlands; Northeastern University, UNITED STATES

## Abstract

A Sequence Recall Task with disyllabic stimuli contrasting either for the location of prosodic prominence or for the medial consonant was administered to 150 subjects equally divided over five language groups. Scores showed a significant interaction between type of contrast and language group, such that groups did not differ on their performance on the consonant contrast, while two language groups, Dutch and Japanese, significantly outperformed the three other language groups (French, Indonesian and Persian) on the prosodic contrast. Since only Dutch and Japanese words have unpredictable stress or accent locations, the results are interpreted to mean that stress “deafness” is a property of speakers of languages without lexical stress or tone markings, as opposed to the presence of stress or accent contrasts in phrasal (post-lexical) constructions. Moreover, the degree of transparency between the locations of stress/tone and word boundaries did not appear to affect our results, despite earlier claims that this should have an effect. This finding is of significance for speech processing, language acquisition and phonological theory.

## Introduction

### Background

In addition to vowels and consonants, the words of a language may be specified for stress and tone. The presence of such word prosodic structure may have profound consequences for speech processing as well as first and second language acquisition [1]. Stress in languages like English is an obligatory syllabic prominence feature of major class words [2]. Such words can be grammatical utterances with or without additional unstressed function words. For instance, *An elephant* is a legitimate answer to a question like *What can you see*?, while *Do it*! is a legitimate imperative. Here, the stressed syllables *el* and *Do* in addition serve as anchor points for elements of the intonational melody known as ‘pitch accents’. In longer utterances, many stressed syllables are spoken without pitch accents, only one of which needs to occur in any utterance. In *ELephants can NO longer be made to perform in CIRcuses*, pitch accents may occur on the capitalized syllables, while *long*, *made* and *form* are stressed syllables that may be left without. Lexical searches during speech processing appear to be initiated at each stressed syllable [3][4], while [5] established the role of pitch in signaling word-initial stress in German. Since stressed syllables may be pitch accented, word stress is indirectly involved in signaling different focus meanings in English and many other languages (e.g. [6][7]). Also, accented words are processed faster than unaccented words [1(p.243)].

Equally, languages may have a tone contrast on one or more syllables in a word. The variation in the density of lexical tones has given rise to sub-classifications of languages with lexical tone, such as ‘restricted tone language’, ‘contour tone language’, ‘pitch accent language’, and so on [2]. Regardless of such variation in tone density, lexical tone, like lexical stress, will play significant roles in speech processing. In Nigerian English, word boundaries are generally marked by pitch features. Specifically, any utterance-medial major class word is ended by a drop in pitch, and it is therefore also begun by a drop in pitch if another major class word precedes; a preceding function word will cause a major class word to begin with a rise [8]. Japanese has a lexical tone melody on one of the syllables of some morphemes (‘accented words’); toneless words are ‘unaccented’. Because of the effect on word beginnings, the distinction between initially accented words and other words is already detected on the basis of the first syllable, while word priming is sensitive to agreement in accentuation [9].

The acquisition of a native word prosodic structure has obvious consequences for foreign language learning later in life. French words have a pitch accent on their final syllable when they appear finally in a phonological phrase, as evidenced by the alignment of the pitch peak in relation to the duration of that syllable [10], while word-initial syllables may equally have a pitch accent when occurring initially in the phonological phrase. Since the location and the presence of the pitch accent is determined by the phrase, lexical representations of French words do not need to register any word prosodic feature. The acquisition by French learners of languages with contrastive word stress will therefore be hampered by the need to register a prosodic feature for which their lexicon was not kitted out. Similarly, while infants appear to start from the assumption that the pitch pattern of words is part of their representation, those acquiring a language without tone abandon that assumption around the age of 9 months [11]. Adult learners of tone languages whose native language has no lexical tone will therefore initially be unable to store the tone pattern of words in an L2 language with lexical tone.

An interesting approach to uncovering the presence of word prosodic structures was developed in a series of experiments by Emmanuel Dupoux and colleagues, who showed that sensitivity to word prosodic contrasts varies considerably with the language background of the listener [12][13][14][15]. While the details of the experimental tasks differ across experiments, they used a sequence order recall task in which trials consist of short sequences of word-like stimuli representing two types depending on the location of the prosodic prominence, for instance [númi] and [numí]. The task involves a reproduction of the order of the stimuli as a sequence of key strokes, whereby key ‘1’ is associated to one stress location (e.g. [númi]) and key ‘2’ to the other (e.g. [numí]). A trial may be from 2 to 6 stimuli in length. Thus, the order [numí—numí—númi] is to be reproduced as ‘221’. While stimuli are unique and thus differ acoustically from one another, they come from two sets whose members are exemplars of each of the two stress patterns. Importantly, it was found that listeners whose native language is French performed significantly worse than Spanish listeners in reproducing the stress patterns by key strokes [12]. A crucial consideration for the interpretation of the language effect concerns the amount of time that elapses between stimulus and response. An AX discrimination task using similar stimuli did not yield any marked difference between French and Spanish listener groups, because subjects can apparently respond quickly enough to be able to rely on their acoustic memory [16]. An ABX task did reveal differences between groups, both in error rates and reaction times, but it was suggested that a Sequence Recall Task (SRT), where sequences of stimuli can exceed three, would more effectively show the language effect [12]. Rather than an ability to perceive the prosodic contrast, the issue therefore appears to be the ability to *store* prosodic features for the duration of the response time. To enhance this dependence on storage, a distracting sound is played after each sequence, in an attempt to inhibit participants’ recourse to acoustic memory. The authors used the term “deafness” to ‘designate the effect of listeners having difficulties in discriminating non-words that form a minimal pair in terms of certain non-native phonological contrasts’ [13], with quotation marks to indicate that the listeners involved do not completely fail to perceive these contrasts.

Our interest in this report concerns the nature of this storage. Dupoux and colleagues discuss three manners in which the information in stimuli could in principle be encoded in a perception task [12]. The first relies on a categorization of the incoming stimulus on the basis of a comparison of its acoustic characteristics with stored exemplars. Robust acoustic cues to stress position should aid subjects in the use of this strategy, but it has the disadvantage of not being automated and thus being hard to apply when stimulus sequences are long and intervals between stimuli are brief. A second strategy they envisage is the use of the Mismatch Negativity signal which is generated in the brain whenever a stimulus differs from the preceding one. The authors briefly contemplate how this signal might be used to classify stimuli as ‘same’ or ‘different’, but, quite apart from its feasibility, reject it as unusable in cases where there are phonetic differences between stimuli that fall within the same stress location category. Their third possibility is an encoding strategy based on phonological representations, which they see as the only plausible route, provided that there is phonetic variation among the stimuli in the same stress location class and that some provision is made in the experiment to defeat acoustics-based strategies. Assuming this third option is correct, the issue concerns the nature of the phonological representation.

Despite their lack of success in the SRT, French listeners are undoubtedly able to phonologically encode a pitch accent on some syllable, since these routinely occur at the beginnings and ends of phonological phrases, as explained above [17][18]. This suggests that the distinction between the French and Spanish listeners lies in whether stress is marked in their lexicon. This position was taken by Sharon Peperkamp [19], who assumes that the presence of lexical markings provides the crucial determinant of success in the SRT. However, Peperkamp’s position differs from ours in that she attributed the presence of prosodic markings to early language acquisition, in particular the stage before word recognition. The argument here is that children cannot detect words if their understanding of the relation between word boundaries and stress location is incomplete. Languages with transparent regularities between stress location and word boundaries, like French, will allow infants to acquire a default stress rule, but—in Peperkamp’s view—both exceptional stress and morphologically induced stress will cause infants to develop (partly redundant) stress marking throughout the lexicon. Exceptional stress is common in languages with default penultimate stress and monosyllabic words. Polish, for instance, overwhelmingly has penultimate stress, but also has a number of words with antepenultimate stress, like *uniˈwersytet* ‘university’, besides a large number of monosyllabic words. Morphologically governed stress generously occurs in Spanish, which also has lexical exceptions. We refer to Peperkamp’s position as the Surface Transparency Hypothesis (STH).

An inherent assumption in the STH is that the adult grammar includes the traces of assumptions that may have been abandoned during the acquisition process. However, developmental studies frequently report perceptual reorganizations as a result of continued exposure to the language being acquired [20]. As observed earlier, the initial hypothesis by infants acquiring Dutch is that words are specified for tonal information, but by 9–12 months they abandon their reliance on pitch, as shown by their results in tone discrimination tasks. By the age of 18 months, their sensitivity increases again so as to reach an adult level of performance, indicative of their acquisition of the intonation system [11]. We therefore assume that if the *adult lexicon* does not contain prosodic markings, the performance of speakers on a SRT will fall short of that by other speakers, regardless of whether that language has a surface-observable relation between stress location and word boundaries.

Persian may provide an opportunity to throw some light on this issue. The surface phonology of Persian presents many cases of phonological word boundaries which fail to correspond to morphological word boundaries. If the infant relies on the detection of these boundaries in the search for lexical items, as suggested by [21], it will be faced with an array of accent locations at various removes from the final boundary. This non-transparent relation between phonological word boundaries and what we here refer to as accent arises due to complexities in the mapping between morpho-syntactic structure and phonological structure. *Morphological words*, whether simple, derivationally complex or compound, are accented on the final syllable. However, prosodically deficient morphemes are integrated into a *phonological word* with a morphological host to their left (cf. [22]). Differently from derivational and inflectional suffixes, these cliticizing morphemes do not form single morphological words with their host (see S1 Text). Effectively, this represents a case of Peperkamp’s morphological stress: ‘As for stress systems in which morphology plays a role, they surely cannot be acquired pre-lexically, since pre-lexical infants do not have access to morphological information by definition’ [19(p.101)]. Strikingly, Persian minimal pairs of word and word+clitic combinations are highly frequent, like /mɒhi/ [mɒ.hí] ‘fish’ and /mɒh-i/ [mɒ́.hi] ‘any/some month’. Minimal pairs may also exist at the level of the phrase due to the status of compounds as single morphological words. Thus, the bi-phrasal NP-VP clausal structure [fɒrsí zabɒ́n ast] (Persian-language-is) ‘Persian is a language’ contrasts with [fɒrsi zabɒ́n ast] ‘S/he is a speaker of Persian’, where [fɒrsi zabɒn] is a compound. In addition, there are further post-lexical accent rules not discussed in detail here, which place accent at the beginning of syntactic constituents, further complicating a direct mapping of accents and phonological boundaries. Yet, because no prosodic marking in the lexicon is required to generate or interpret Persian sentences, our prediction is that listeners with Persian as their L1 are stress “deaf”.

We refer to the Persian post-lexical prominence as ‘accent’, because its ‘stress’ (e.g. [23]) does not involve any phonetic cues, like durational and spectral features, other than f0, and technically therefore the prosodic marking is tonal, as in Japanese [24][25][26]. Results obtained with a SRT for a group of participants with a Standard Japanese background and a group of speakers of an accentless variety of Japanese indicate that the presence of a lexical accent allows participants to perform the SRT successfully [27]. The standard group significantly outperformed the accentless group on the prosodic contrast corresponding to lexical accent differences in Standard Japanese. Our assumption is therefore that either prosodic marking, stress or accent (i.e., tone), will enable participants to perform successfully on the SRT.

### Languages in the experiment

In order to put the performance of Persian listeners in perspective, we selected upper and lower baseline languages. The first two rows in Table 1 list the phonetic parameters that cue the word prominence, row 3 indicates whether the prosodic feature is present on all words, row 4 indicates whether the adult grammar requires any words to be marked in the lexicon for the presence of the stress or tone, row 5 indicates whether the prosodic marking has a stable relation to observable word boundaries in the sense of Peperkamp [19], implying a prediction of relative insensitivity to prosodic contrasts by the STH if the answer is ‘yes’. For Indonesian, which we assume has no prosodic feature on any syllable, a situation not envisaged in [19], there is technically no prediction by the STH, but it is reasonable to assume the language would by default have induced stress “deafness” in her account. Row 6 presents the prediction by our hypothesis based on a marking in the adult lexicon.

**Table 1:** Word prosodic features of five languages in the experiment (rows 1–5) and predictions for success in the Sequence Recall Task (row 6).

	Dutch	Japanese	Persian	French	Indonesian
1. Duration/spectral	Yes	No	No	No	No
2. Tonal	Yes, if accented	Yes	Yes	Yes	No
3. Obligatory	Yes, stress	No	Yes; deletable	Yes; deletable	-
4. Lexical	Yes, stress	Yes, tone	No	No	-
5. Surface transparent?	No	No	No	Yes	-
6. Stress “deaf” (this paper)?	No	No	Yes	Yes	Yes

For the lower baseline languages, we chose French and Indonesian. French has phrase-peripheral pitch accents, a transparent relation between accents and boundaries, and is a textbook case for stress “deafness”. Indonesian has neither tone or stress on any syllable, whether word-based or phrase-based [28][29]. The performance of listener groups with these two language backgrounds should provide an operational definition of stress “deafness” as defined by the SRT. For the upper baseline, we chose Dutch and Japanese. Dutch has numerous exceptional stress locations, as illustrated by minimal pairs like [ˈkaːnɔn] ‘canon’—[kaˈnɔn] ‘cannon’ [26]. Japanese words unpredictably fall into two classes, unaccented and accented, with free accent location, a minimal triplet being [hási] ‘chopsticks’, [hasí] ‘bridge’ and unaccented [hasi] ‘end’ [30][31]. The language under investigation is Persian, which has a non-transparent relation between perceivable word boundaries and accent location, while not requiring lexical prosodic markings in the adult grammar.

Our SRT broadly followed those of the more recent publications [12][13][14][15]. One innovation concerns the language in which the stimuli are spoken. In the earlier experiments this was Dutch, which has word stress. Since Persian lacks phonetic stress, as explained above, we included stimuli spoken in Dutch and Persian. Our stimuli thus always contained the prosodic feature present in Dutch (stress), French (pitch accent), Japanese (pitch accent) and Persian (pitch accent). As in [12], the recall performance of a prosodic contrast is compared with that of a control segmental contrast across different levels of memory load. The experiment is divided into two parts in each of which participants are required to learn two CVCV nonwords representing either a segmental contrast (e.g. [múku—múnu]) or a prosodic feature contrast (e.g. [númi—numí]).

In order to make participants tap into a phonological level of representation, three measures can be taken. First, stimuli representing each phonological type should vary phonetically, so that the participants cannot easily use low-level acoustic cues. In our case, each phonological type was represented by three acoustically different tokens. Second, to further minimize the use of non-linguistic coding strategies, we kept stimulus durations and inter-stimulus intervals fairly short, at 450 ms and 120 ms, respectively. Third, immediately after playing each sequence the word ‘OK’ was played. These features make it unlikely that participants can rely on ‘echoic memory’, the ability of the brain to take a copy of what is heard and hold it for 2 to 5 seconds [32].

We avoided mixing stimuli from different speakers in the same sequence, unlike the procedure of the ABX tasks in [16] and in the SRTs in [12] and [14]. These authors motivated this procedure on the grounds that it made the task more difficult and hence more likely to show up differences between listener groups. In a pilot experiment we found that using multiple voices for the same sequence was highly disturbing for the participants. Moreover, we observed that mixing speakers seemed to have opposite effects, depending on the combination of speaker and prosodic pattern. Using one voice for the first and second stimuli in the sequence [númi—númi—numí—numí] and another for the third and fourth, for instance, makes it easier to spot the shift from initial to final stress, because the prosodic difference is highlighted by the speaker difference.

## Materials and Methods

### Materials

Two minimal pairs of non-words were constructed, one involving a segmental contrast ([múku—múnu]) and a prosodic contrast ([númi—numí]). None of the nonwords is a real word in Persian, Dutch, Japanese, French or Indonesian, while being phonotactically legal combination of segments in all of these languages. These nonwords were recorded several times by a female and a male speaker of Persian and of Dutch, respectively, in a sound-proof booth, at a sampling rate of 22050 Hz. In addition, the word ‘OK’ was recorded by a different female speaker of Persian. For each nonword, three tokens from each speaker were selected that were judged by the authors to clearly illustrate the contrasts under investigation. This yielded 48 stimuli (4 nonwords × 4 speakers × 3 tokens). Mean durations were 581 ms (Persian segmental), 583 ms (Persian prosodic), 463 ms (Dutch segmental) and 452 ms (Dutch prosodic). Using the PSOLA algorithm implemented in Praat [33], durations of all tokens were changed to 450 ms, a shortening which preserved the language-like nature of the stimuli. Acoustic details of the stimuli representing the prosodic contrast are given in Table 2.

**Table 2:** Mean acoustic measurements of the prosodic tokens after durational adjustments pooled over 6 tokens of each nonword ([númi / numí]) for Persian and Dutch separately.

	1^st^ word [númi]		2^nd^ word [numí]	
	Dutch set	Persian set	Dutch set	Persian set
Dur. σ1 (ms)	215	210	195	180
Dur. σ2 (ms)	235	240	255	270
Dur. (σ1/σ2)	0.91	0.87	0.76	0.67
Int. σ1(dB)	86.3	86.6	83	85.1
Int. σ2 (dB)	77.4	78.6	86.4	86.7
Int. (σ1/σ2)	1.11	1.11	0.96	0.98
F0_Max_ σ1(ST)^a^	10.82	11.25	3.45	6.83
F0_Min_ σ1 (ST)	6.41	6.88	1.01	4.88
F0_Max_ σ2 (ST)	3.07	7.27	9.62	8.60
F0_Min_ σ2 (ST)	-0.09	3.13	0.81	4.48
F0 (σ1/σ2)	1.39	1.05	0.27	0.47
F1 (V1/V2)^b^	1.11	1.24	0.94	1.00
F2 (V1/V2)	0.46	0.57	0.44	0.60
F3 (V1/V2)	0.70	0.85	0.72	0.86

Dur. σ1: duration of the 1^st^ syllable; Dur. σ2: duration of the 2^nd^ syllable; Dur. (σ1/σ2): ratio between the duration of the 1^st^ syllable and the duration of the 2^nd^ syllable; Int. σ1: intensity of the 1^st^ syllable; Int. σ2: intensity of the 2^nd^ syllable; Int. (σ1/σ2): ratio between the intensity of the 1^st^ syllable and the intensity of the 2^nd^ syllable; F0_Max_ σ1: maximum F0 of the 1^st^ syllable; F0_Min_ σ1: minimum F0 of the 1^st^ syllable; F0_Max_ σ2: maximum F0 of the 2^nd^ syllable; F0_Min_ σ2: minimum F0 of the 2^nd^ syllable; F0 (σ1/σ2): ratio between the pitch range of the 1^st^ syllable and the pitch range of the 2^nd^ syllable; F1 (V1/V2): ratio between the 1^st^ formant of the 1^st^ vowel and the 1^st^ formant of the 2^nd^ vowel; F2 (V1/V2): ratio between the 2^nd^ formant of the 1^st^ vowel and the 2^nd^ formant of the 2^nd^ vowel; F3 (V1/V2): ratio between the 3^rd^ formant of the 1^st^ vowel and the 3^rd^ formant of the 2^nd^ vowel.

^a^ Reference value for semitone calculations was 100 Hz.

^b^ Formant frequencies were extracted from the midpoints of each vowel at the highest intensity peak, using a linear predictive coding algorithm in Praat.

The study employed a repeated measures design, with LANGUAGE as the fixed between-participant factor, and CONTRAST, SEQUENCE LENGTH and STIMULUS TYPE as the fixed within-participant factors [5×2×3×2: LANGUAGE (Persian, Dutch, Japanese, French and Indonesian) × CONTRAST (segmental and prosodic) × SEQUENCE LENGTH (3-, 4- and 5-word) × STIMULUS TYPE (Persian set and Dutch set)].

### Procedure

The experiment was presented with E-Prime 2.0 [34] on a laptop computer. Participants listened individually to the stimuli through loudspeakers in an otherwise soundless room. The language of the experiment was English for all language groups. Instructions were provided both on the screen (in English) and in printed form (in each native language). The experiment consisted of two parts, one for the segmental contrast and one for the prosodic contrast, with a voluntary break in between. Each part was preceded by a training session. For the segmental test, participants were trained to associate nonword [múku] with key ‘1’ and [múnu] with key ‘2’, while for the prosodic test they were trained to associate [númi] with key ‘1’ and [numí] with key ‘2’. Participants were told that they were going to learn two words in a foreign language and were invited to press key ‘1’ so as to hear all 12 tokens of one member of the contrast and key ‘2’ for all 12 tokens for the other member. Next, they were invited to listen to a single, randomly presented token from each set by pressing either key. In this way, listeners could hear the various tokens of the two words as often as they wished. After they had indicated having learned this two-way classification, participants moved on to an identification task. At this stage, they heard one token from the 24 stimuli and were asked to respond by pressing ‘1’ or ‘2’, after which either the word “CORRECT!” or “INCORRECT!” was displayed on their screen for 800 ms. This procedure was repeated until eight correct sequential responses had been given. Maximally two stimuli for the same word from each language set were played in succession. After passing this training session, participants entered the experimental session. In each language group, half of the participants were first tested with the segmental contrast.

During the experimental session, participants first listened to a warm-up block of two-word sequences and were asked to reproduce each sequence by typing the associated keys in the correct order. It contained all four possible combinations (‘11’, ‘12’, ‘21’ and ‘22’), which resulted in eight trials (4 sequences × 2 language sets). After any incorrect response, the sequence was repeated until the correct response was provided.

In the test block, we used sequences with three, four and five words, each containing five different combinations of the two words. The choice of these combinations was a compromise between a maximum number of switch points (transitions from ‘1’ to ‘2’ or from ‘2’ to ‘1’) and an avoidance of regular switch patterns (i.e. ‘12121’). For three-word sequences, there are two combinations with two switch points, both of which were used, and four possible combinations with one switch point, three of which were used. In four-word sequences, we used five combinations with two switch points, out of the six that are possible. For the five-word sequences, we chose five out of the eight possible patterns with three switch points. The selected sequences are given in Table 3. There were 10 trials (5 sequences × 2 language sets) per sequence length, which resulted in overall 30 trials.

**Table 3:** Sequences of nonwords used in the experiment.

Three-word sequences				
112^a^	121	122	211	212
Four-word sequences				
1211	1221	2112	2122	2212
Five-word sequences				
12112	12122	12212	21211	21221

^a^ 1 = first nonword, 2 = second nonword.

In the test block, there was no feedback on responses. The order of the sequences within the blocks was randomized per subject. Within each sequence, the items were randomly instantiated by one of the three tokens from either female speaker or male speaker, but no token appeared more than once per sequence. Tokens were separated by 120 ms intervals. Responses could not be given until a recording of ‘OK’ of 450 ms had been played 120 ms after the offset of the last token in the sequence. Once a response was given, the participants had to confirm it by pressing the Enter key, after which there was a 1500 ms interval till the next sequence was played. Whenever a sequence was entered that didn’t match input sequence length, participants were asked to enter the response again. On average, the entire experiment lasted about 25 minutes for each participant.

### Participants

150 participants took part in the experiment. They were all university students or held recently obtained MA degrees. The mean age for Persian, Dutch, Japanese, Indonesian and French was 27 (SD = 7), 27 (SD = 4), 21 (SD = 3), 27 (SD = 4) and 29 (SD = 8), respectively. None of the participants had stayed in a foreign country for more than 18 months, nor had they had any professional musical training, which might have facilitated this prosodic SRT [35]. The Persian participants were recruited in Tehran and tested at the University of Tehran, the Japanese participants were recruited in the Tokyo area and tested at Waseda University, the Dutch participants were recruited in Nijmegen and tested at Radboud University Nijmegen, while the French participants were from the Provence-Alpes-Côte d'Azur region and were tested at Laboratoire Parole et Langage (LPL) in Aix-en-Provence. Half of the Indonesian participants, all of whom were fluent in standard Indonesian, were recruited in the Netherlands and tested at Radboud University Nijmegen, while the other half were recruited and tested at the University of Muhammadiyah Malang in East Java. The average number of trials participants needed to pass the training identification task and the warm-up block was 40. The scores of one French and three Persian listeners were discarded because they produced more than 150 incorrect responses in the warm-up block. Four new subjects (one French and three Persian) were tested at Radboud University Nijmegen.

### Ethics Statement

The study was approved by Ethics Assessment Committee of the Faculty of Arts at Radboud University Nijmegen, and written consent was obtained from participants. All participants were paid a small fee for their participation.

## Results

Dupoux and colleagues normalized the stress contrast scores relative to a baseline by subtracting the scores of the segmental contrast from them [12], while in an earlier study they had processed the stress contrast directly [16]. These authors discussed some arguments for and against the baseline method [12]. An argument they raise against it is that difference scores should have inherent measurement errors that are twice the size of those of absolute scores and are for this reason less reliable. However, according to [36], our procedure falls within the great majority of experimental conditions in which difference scores are quite reliable. We chose to use absolute scores, because we have a relatively large and homogeneous participant group, 30 in our case, against 12 in [12]. Also, we failed to find any significant differences between participant groups on the segmental contrast, which is an indication that there was no need for this baseline in the analysis of the prosodic contrast scores. By not using difference scores, we moreover avoided a decision on what the optimally language-neutral choice for the segmental contrast should be. With the minimal pairs we used, based on [15], we have no *a priori* guarantee that the status of this particular segmental contrast is equivalent across languages.

Responses that were fully correct transcriptions of the input sequence were labelled CORRECT, while all other responses were labelled INCORRECT. Tables 4, 5 and 6 give score values for each language group as a function of contrast and stimulus type at the 3-word, 4-word and 5-word levels of sequence length, respectively. We had no missing data.

**Table 4:** Mean scores (percentages correct) for each language group as a function of contrast and stimulus type at the 3-word level of sequence length.

	Segmental contrast		Prosodic Contrast	
	Dutch Set	Persian Set	Dutch Set	Persian Set
Persian	91.33 (16.34)^a^	94.00 (11.92)	67.33 (30.84)	61.33 (31.92)
Dutch	96.00 (9.68)	98.00 (6.10)	69.33 (28.15)	82.00 (23.69)
Japanese	96.67 (9.22)	96.00 (12.20)	82.67 (22.12)	78.67 (23.45)
Indonesian	92.67 (15.30)	94.00 (14.04)	60.67 (35.42)	50.67 (37.78)
French	95.33 (15.48)	94.67 (11.66)	67.33 (29.93)	55.33 (32.67)

^a^ Standard deviations are given in parentheses.

**Table 5:** Mean scores (percentages correct) for each language group as a function of contrast and stimulus type at the 4-word level of sequence length.

	Segmental contrast		Prosodic Contrast	
	Dutch Set	Persian Set	Dutch Set	Persian Set
Persian	84.67 (17.17)^a^	76.67 (25.23)	42.67 (33.52)	42.67 (30.50)
Dutch	88.67 (14.56)	92.00 (14.48)	68.67 (32.67)	66.0 (32.01)
Japanese	86.67 (16.04)	84.0 (22.53)	62.67 (30.05)	71.33 (27.13)
Indonesian	77.50 (23.52)	81.33 (20.96)	51.33 (34.31)	38.0 (34.18)
French	81.33 (22.85)	80.67 (24.90)	44.0 (29.90)	39.33 (29.93)

^a^ Standard deviations are given in parentheses.

**Table 6:** Mean scores (percentages correct) for each language group as a function of contrast and stimulus type at the 5-word level of sequence length.

	Segmental contrast		Prosodic Contrast	
	Dutch Set	Persian Set	Dutch Set	Persian Set
Persian	67.33 (29.0)^a^	74.67 (26.23)	32.00 (25.51)	26.00 (24.72)
Dutch	77.33 (19.46)	78.00 (21.88)	46.00 (28.36)	51.33 (33.50)
Japanese	73.33 (24.82)	75.33 (20.80)	53.33 (24.82)	49.33 (24.48)
Indonesian	64.67 (27.13)	64.67 (30.03)	32.00 (26.57)	29.33 (35.13)
French	66.67 (27.46)	62.00 (26.44)	28.00 (30.89)	22.67 (24.48)

^a^ Standard deviations are given in parentheses.

These data were subjected to a repeated measures ANOVA with the between-participant factor LANGUAGE (Persian, Dutch, Japanese, Indonesian, French) and three within-participant factors CONTRAST (segmental, prosodic), SEQUENCE LENGTH (3-word, 4-word, 5-word) and STIMULUS TYPE (Persian set, Dutch set). We applied arcsine transformations prior to analysis, since the variances of distributions underlying percentages were not constant and the unit of proportions was not constant over the scale (see [37(p.134)]). In all analyses, Huynh-Feldt corrected p-values are reported where appropriate. The ANOVA is summarized in Table 7. As for the within-participant factors, the analysis revealed significant main effects of CONTRAST (*p* <.001, $\eta_{\text{p}}^{\text{2}}$ = .685), and SEQUENCE LENGTH (*p* <.001, $\eta_{\text{p}}^{\text{2}}$ = .689), with relatively large effect sizes. We found no significant main effect for STIMULUS TYPE (*p* = .292). Overall, participants performed substantially worse in the prosodic condition than in the segmental condition and longer sequences yielded more errors than shorter ones. Participants in all language groups performed above chance level for both the segmental and the prosodic contrast.

**Table 7:** Summary of the repeated measures ANOVA: Scores by the language of the listener, the type of the contrast, the length of the sequence and the type of the stimulus.

Effects	Sum of squares	*df*	Mean squares	*F* value	*P* value	$\eta_{p}^{2}$
LANGUAGE (L)	67.51	4, 145	16.88	6.72 *	**<.001**	.156
CONTRAST (C)	346.65	1, 145	346.65	315.71 *	**<.001**	.685
SEQUENCE LENGTH (SL)	222.09	2, 290	111.04	320.88 *	**<.001**	.689
STIMULUS TYPE (ST)	0.42	1, 145	0.42	1.12	.292	.008
C × L	19.41	4, 145	4.85	4.42 *	**.002**	.109
SL × L	5.24	8, 290	0.65	1.89	.061	.050
ST × L	4.00	4, 145	1.00	2.66 *	**.035**	.068
C × SL	0.79	2, 290	0.40	1.10	.334	.008
C × SL × L	1.92	8, 290	0.24	0.66	.722	.018
C × ST	1.21	1, 145	1.21	4.07 *	**.046**	.027
C × ST × L	2.16	4, 145	0.54	1.81	.131	.047
SL × ST	0.06	2, 290	0.03	0.09	.913	.001
SL × ST × L	2.32	8, 290	0.29	0.87	.542	.023
C × SL × ST	0.38	2, 290	0.02	0.06	.938	.000
C × SL × ST × L	4.55	8, 290	0.57	1.94	.055	.051

* indicates a significant effect at the 5% level.

**Fig 1:**
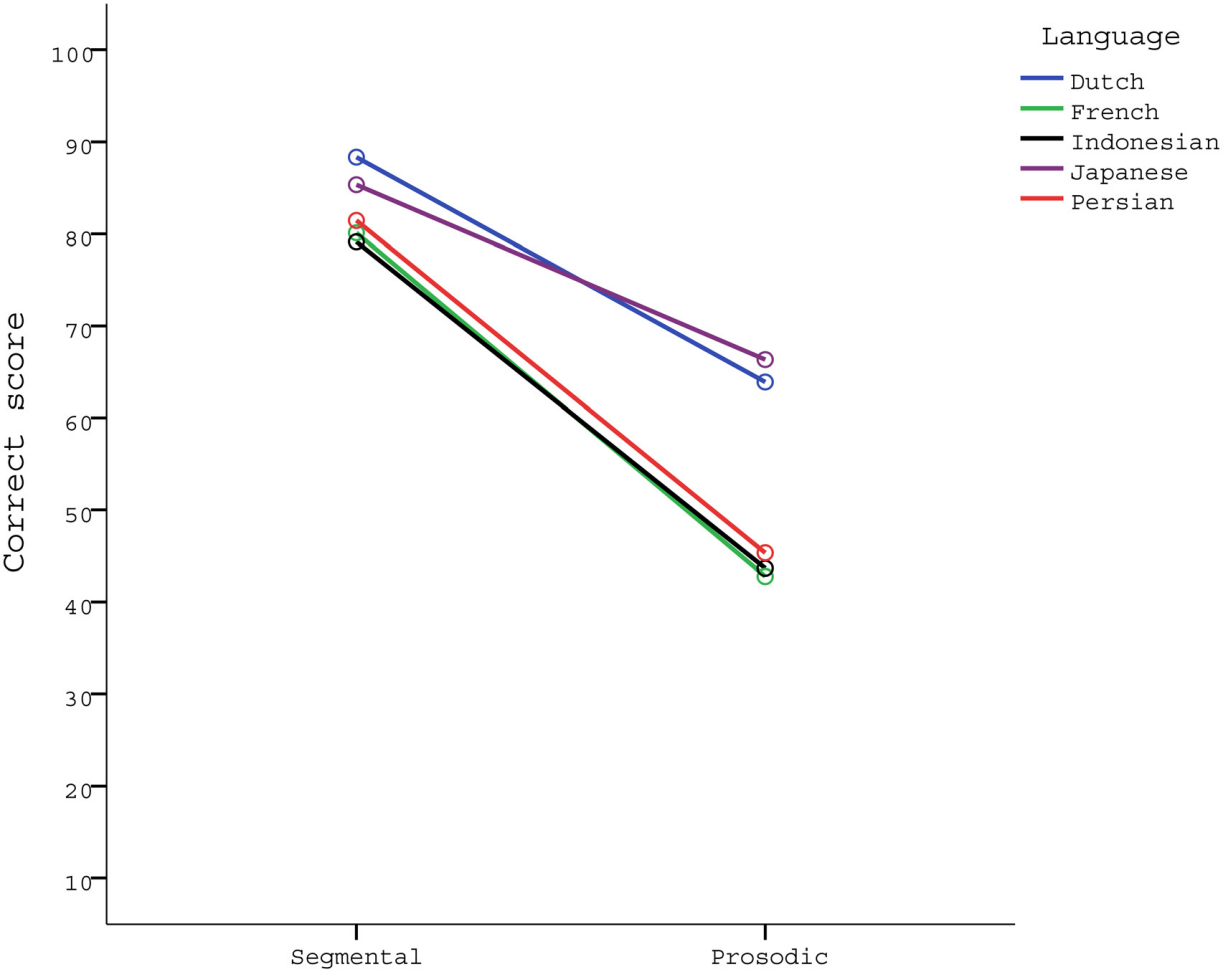
Mean scores for each language group across the two contrasts.

The between-participant factor, LANGUAGE, was significant (*p* <.001, $\eta_{\text{p}}^{\text{2}}$ = .156), while there was a significant interaction between CONTRAST and LANGUAGE (*p* = .002, $\eta_{\text{p}}^{\text{2}}$ = .109). All other significant interactions, i.e., STIMULUS TYPE × LANGUAGE and CONTRAST × STIMULUS TYPE, produced very small effect sizes ($\eta_{\text{p}}^{\text{2}}$ <.100), suggesting that they are unimportant for purposes of this study. Therefore, in the analysis that follows we collapsed over SEQUENCE LENGTH and STIMULUS TYPE in each language group. (But see S2 Text for the analyses of the different sequence lengths and stimulus types as well as an alternative overall analysis.) Fig 1 gives mean score values for each language group across the two contrasts (pooled over the three sequence lengths and the two stimulus types).

Given the significant interaction between CONTRAST and LANGUAGE, we carried out separate one-way ANOVAs of each of the two contrasts to investigate the difference between languages at each level. The analyses are reported in Table 8. Results revealed that the difference between the languages was significant only in the prosodic contrast (*p* <.001, $\eta_{\text{p}}^{\text{2}}$ = .174).

**Table 8:** Summary of the separate one-way ANOVAs for the segmental and prosodic contrasts.

	Sum of squares	Mean squares	*F* (4, 145)	*P* value	$\eta_{p}^{2}$
Segmental contrast	1.38	0.34	2.00	.098	.052
Prosodic contrast	13.76	3.26	7.63 *	**<.001**	.174

* indicates a significant effect at the 5% level.

A post-hoc Sidak test yielded two homogeneous sets, one with Dutch and Japanese and one with Persian, French and Indonesian. Table 9 summarizes the result. Overall, Japanese and Dutch participants performed better at the prosodic contrast, while French, Indonesian and Persian participants performed worse.

**Table 9:** Summary of a one-way ANOVA with a Sidak post-hoc analysis for the prosodic contrast.

Language (I)	Language (J)	Mean Difference (I-J)	*P* value	95%CI
Persian	Dutch	-0.53 *	**.021**	[-1.01, -0.05]
	Japanese	-0.57 *	**.009**	[-1.05, -0.09]
	Indonesian	0.07	1.000	[-0.41, 0.55]
	French	0.07	1.000	[-0.40, 0.55]
Dutch	Japanese	-0.04	1.000	[-0.52, 0.43]
	Indonesian	0.60 *	**.005**	[0.12, 1.08]
	French	0.60 *	**.005**	[0.12, 1.08]
Japanese	Indonesian	0.64 *	**.002**	[0.16, 1.12]
	French	0.65 *	**.002**	[0.17, 1.13]
Indonesian	French	0.01	1.000	[-0.47, 0.49]

* indicates a significant difference at the 5% level.

Since LANGUAGE is only significant for the prosodic contrast, we carried out separate one-way ANOVAs for the prosodic contrast for the Dutch and Persian stimulus sets, which in both cases yielded significant effects of LANGUAGE, with marginally more discrimination produced by the Persian stimulus set (Dutch stimulus set: *F*(4,145) = 3.98, *p* = .004, $\eta_{\text{p}}^{\text{2}}$ = .099; Persian stimulus set: *F*(4,145) = 9.62, *p* <.001, $\eta_{\text{p}}^{\text{2}}$ = .210). In a Sidak post-hoc analysis, we found two homogeneous groups of languages, Dutch and Japanese in one set and Persian, French and Indonesian in the other, for the Persian stimulus set.

## Discussion

Our Sequence Recall Task (SRT) experiment with 150 subjects equally divided over five participant language groups produced results that support a number of positions we have taken in the introduction. The finding that speakers of Persian performed as poorly as the French listeners despite the omnipresence of accent location contrasts in the surface phonology of their language supports the position that the crucial determinant of success in the SRT is the presence of prosodic markers in the lexicon. It also supports our position, *contra* [19], that the degree of transparency in the relation between perceivable word boundaries and accent location is not relevant, as long as the adult grammar operates without any lexical markings. Earlier, Peperkamp [19] had proposed that the relation between prosodic stress and word boundaries should be transparent to the degree it is in French, where pitch accents predictably occur on final and initial syllables of phrases, or in languages with an exceptionless stress rule that alternates between the word final and word-penultimate position depending of the vowel length of the final syllable, like Hawaiian, or *a fortiori* in languages in which an audible stress is present at every audible word boundary. While we do not exclude that Persian learning infants might initially provide their phonological representations of words with an accent, such initial stages would not survive in the adult grammar. The flexibility of the grammar during acquisition contrasts with its consolidation during adulthood, as shown by the poor results of L2 learners of Tokyo Japanese, a language with lexically contrastive pitch accents, by speakers of accentless Japanese dialects [27][38].

The lexical representational basis of the successful completion of the SRT is emphatically supported by the failure of speakers of Persian to perform at the level of speakers of languages with lexically contrastive prosodic features, like Dutch and Japanese. Speakers of Persian are widely exposed to the *post-lexically* contrastive function of accent in their language and will immediately notice incorrect accent placements. The prosody of Persian is governed by the morpho-syntax rather than the phonology. Peperkamp’s account of surface-transparent strategies to word detection by infants may well be realistic for the earlier stages of language acquisition, but what counts for the adult language user is the status of the grammar as it developed to its final state. Persian infants will at some early point come to realize that configurations of audible word boundaries and accent locations can fruitfully be used for word detection. At some point they will discover that (ignoring syntax-induced initial accents) the stretch between a boundary and a preceding accent contains clitic words, plus or minus any intervocalic syllable-initial consonant in that location (cf. /mɒh-i/ [mɒ́.hi] ‘any/some month’), while the morphological word starts at the preceding boundary and ends at the accent, equally *modulo* the syllabic affiliation of its final consonant. This discovery will inevitably lead to an absence of prosodic markings in their lexicon, and to stress “deafness” during adulthood. The fact that accent has a high functional load in Persian and that deviations in accent locations are very salient to them cannot change this, assuming—as we have argued and as is suggested by the results of our experiment—that it is the structure of the adult lexicon that determines what can be lexically stored.

Our result underscores the significance of the SRT for phonological theory as developed by Emmanuel Dupoux and Sharon Peperkamp. It discriminates between lexical and post-lexical representations, a distinction that is at the heart of the theory of Lexical Phonology [39][40] and its version as developed within Optimality Theory [41][42]. This distinction tends to be blurred in other proposals, like classic Optimality Theory (OT) and the various adaptations that sought to counteract the lack of success it had in dealing with effects that may be attributable to the lexical—post-lexical distinction. The SRT, as our results suggest, appears to provide a robust empirical approach to this distinction and can be used to demonstrate its cognitive basis. Second, our results clearly do not confirm purely episodic models of representation, like radical versions of exemplar theory, confirming [43].

The fact that our results reveal a clean division into two language groups cannot at this point be interpreted to mean that there are no intermediate languages. While the performance of French listeners was generally the lowest, Dupoux and colleagues report fairly strong stress “deafness” results for subjects with Finnish and Hungarian backgrounds, languages with exceptionless word-initial stress. Subjects with a Polish background showed only a marginal effect [13][15], which the authors attribute to loanwords with final or antepenultimate stress, pointing out that a word-final pattern of penultimate stress inherently contrasts with stress on monosyllabic words, which is interpretable as being either initial or final. The presence of this variability may by itself allow speakers of this language to outperform speakers of languages with exceptionless initial or final stress. Recently, listeners with a European Portuguese background, a language for which neither Peperkamp [19] nor this paper would have predicted stress deafness, were shown to be stress “deaf” for stimuli that contain only high vowels, but not for stimuli containing other vowels [44]. Vowel reduction, a correlate of stress in the language for non-high vowels, has apparently taken over the prosodic lexical markings in its speakers, so that they do not respond to prosodic cues if these provide the only difference between the stimuli.

A practical problem in making comparisons between languages is the variation in the details of the experimental designs. One relevant finding in our experiment was the fact that the language in which the stimuli are spoken made no difference to the results for any of the five language groups. Reassuringly, one set was spoken in Dutch, following the use of Dutch stimuli in [12][13][14][15][16], a language with salient phonetic stress marked by an intonational pitch accent, while the other was spoken in Persian, which lacks phonetic stress. This means that the results are neither dependent on the identity of the stimulus language with the language of the subjects nor on the phonological nature of the experimental word prosodic feature. Our experiment is available as S1 File.

## Supporting Information

S1 Dataset The data used in the analysis. (XLSX)

S1 File The experiment file and the audio stimuli. (ZIP)

S1 Text Clitic types. Contains a brief characterization of Persian clitics, with supplemental references [45][46][47].
(DOCX)

S2 Text Supplemental statistics. Contains an alternative analysis in which perfect reversed responses were excluded from the data.
(DOCX)
