# Supplementary material for: Stress “Deafness” Reveals Absence of Lexical Marking of Stress or Tone in the Adult Grammar
Source: PLoS One. 2015 Dec 7;10(12):e0143968. doi: 10.1371/journal.pone.0143968 (PMC4671725; doi:10.1371/journal.pone.0143968)
Supplement: S1 Text — Contains a brief characterization of Persian clitics, with supplemental references [45][46][47]. (DOCX) [file pone.0143968.s003.docx]

**S1 Text. Clitic types**

Three types have been distinguished in the literature, particles, phrasal suffixes and pronominal suffixes. Particles have a variety of functions that represent positions in the syntactic phrase, like auxiliaries, conjunctions and focus governing words like [-am] ‘also’ [45]. The two types of ‘suffixal’ clitics show some inflectional properties, like the indefinite marker [-i] and the object marker [-o], which in some semantic sense are closer to their host and have been called ‘post-lexical suffixes’ [46]. Within this group of post-lexical suffixes, a distinction has been made between those that attach to phrases (‘phrasal suffixes’), like the examples given here, and those that attach to heads, like the pronominal clitics [46]. Particles, phrasal suffixes and pronominal suffixes, all of which begin with a vowel, behave differently when the host word ends in a vowel. Particles tolerate vowel hiatus (e.g. [labu]+[-am]:[labuam] ‘also beetroot’). Phrasal suffixes optionally resolve vowel hiatus by the insertion of a consonant (e.g. [labu]+[-i]:[labuji] or [labui] ‘any beetroot’), while pronominal suffixes avoid vowel hiatus by deleting their initial vowel (e.g. [labu]+[-am]:[labum] ‘my beetroot’). Importantly, all of them share the classic ‘clitic’ criterion of not being particular to the word class of their host [47]. For instance, possessive pronouns will attach to the last word in the simplex NP (e.g. [medɒd-am] ‘my pencil’, [medɒde sabz-am] ‘my green pencil), while personal pronominal attach either to verbs (e.g. [xord-am] ‘I ate’) or to nouns/adjectives (e.g. [xub-am] ‘I am fine’).
