# Supplementary material for: Stress “Deafness” Reveals Absence of Lexical Marking of Stress or Tone in the Adult Grammar
Source: PLoS One. 2015 Dec 7;10(12):e0143968. doi: 10.1371/journal.pone.0143968 (PMC4671725; doi:10.1371/journal.pone.0143968)
Supplement: S2 Text — Contains an alternative analysis in which perfect reversed responses were excluded from the data. (DOCX) [file pone.0143968.s004.docx]

**S2 Text. Supplemental statistics: Analysis with reversals excluded**

Peperkamp and colleagues [15] rejected 32 subjects because they had a disproportionate number of incorrect responses which were perfect reversals of the correct scores, on the ground that these subjects may have confused the keys when typing in their scores. Table 1 gives percentages of perfect reversals of the total number of responses within each of the two tests for each language group separately. The fact that the ratio of the percentages for the segmental and prosodic contrasts (3.62/9.33, or 0.39) is similar to that of the incorrect scores overall (17.14/47.61, or 0.36) suggests that perfect reversals really are incorrect scores, for which reason we treated them as such in the analysis reported in the main text. However, because the Persian participants, a key subject group for our hypothesis, had the highest number of perfect reversals (Table 1) and because the perfect reversals in the prosodic and segmental tests together represented a higher percentage of the total responses (6.47%) than the percentage of theoretically possible responses (which is just under 1%, while granting that many theoretically possible responses never occurred), we decided to perform an alternative analysis on the basis of score values calculated according to (i).

(i)

*Score =* $\frac{CORRECT}{CORRECT+INCORRECT-REVERSED}$ × 100

These data, after arcsine transformations, were subjected to a repeated measures ANOVA with the between-participant factor LANGUAGE (Persian, Dutch, Japanese, Indonesian, French) and three within-participant factors CONTRAST (segmental, prosodic), SEQUENCE LENGTH (3-word, 4-word, 5-word) and STIMULUS TYPE (Persian set, Dutch set). The ANOVA is summarized in Table 2. The analysis revealed significant main effects of CONTRAST (*p*<.001, $\eta_{p}^{2}$=.685), and SEQUENCE LENGTH (*p*<.001, $\eta_{p}^{2}$=.703), with relatively large effect sizes. We found no significant main effect for STIMULUS TYPE (*p*=.211).

The between-participant factor, LANGUAGE, was significant (*p*<.001, $\eta_{p}^{2}$=.157), while there was a significant interaction between CONTRAST and LANGUAGE (*p*=.001, $\eta_{p}^{2}$=.116). All other significant interactions, i.e. SEQUENCE LENGTH × LANGUAGE, STIMULUS TYPE × LANGUAGE and CONTRAST × STIMULUS TYPE, produced very small effects.

**Table 1 – Percentages of perfect reversals by test and language group**

| *Language* | *Segmental* | *Prosodic* |  |
| --- | --- | --- | --- |
| Dutch | 1.33% | 8.33% |  |
| Indonesian | 3.33% | 9.77% |  |
| Japanese | 2.65% | 6.77% |  |
| French | 3.33% | 8.33% |  |
| Persian | 4.5% | 13.44% |  |
| *Average* | 3.62% | 9.33% | 6.47% |

**Table 2. Summary of the repeated measures ANOVA: Scores by the language of the listener, the type of the contrast, the length of the sequence and the type of the stimulus.**

| **Effects** | **Sum of squares** | ***df*** | **Mean squares** | ***F* value** | ***P* value** | $\boldsymbol{\eta}_{\mathbf{p}}^{\mathbf{2}}$ |
| --- | --- | --- | --- | --- | --- | --- |
| LANGUAGE (L) | 64.63 | 4, 145 | 16.16 | 6.76 * | **<.001** | .157 |
| CONTRAST (C) | 321.09 | 1, 145 | 321.09 | 315.66 * | **<.001** | .685 |
| SEQUENCE LENGTH (SL) | 228.04 | 2, 290 | 114.02 | 343.70 * | **<.001** | .703 |
| STIMULUS TYPE (ST) | 0.57 | 1, 145 | 0.57 | 1.58 | .211 | .011 |
| C × L | 19.43 | 4, 145 | 4.86 | 4.77 * | **.001** | .116 |
| SL × L | 5.76 | 8, 290 | 0.72 | 2.17 * | **.030** | .056 |
| ST × L | 3.72 | 4, 145 | 0.93 | 2.59 * | **.039** | .067 |
| C × SL | 1.61 | 2, 290 | 0.80 | 2.36 | .096 | .016 |
| C × SL × L | 2.27 | 8, 290 | 0.28 | 0.83 | .575 | .022 |
| C × ST | 1.49 | 1, 145 | 1.49 | 5.34 * | **.022** | .036 |
| C × ST × L | 2.63 | 4, 145 | 0.66 | 2.35 | .057 | .061 |
| SL × ST | 0.03 | 2, 290 | 0.02 | 0.05 | .954 | .000 |
| SL × ST × L | 2.19 | 8, 290 | 0.27 | 0.85 | .561 | .023 |
| C × SL × ST | 0.13 | 2, 290 | 0.01 | 0.02 | .977 | .000 |
| C × SL × ST × L | 4.34 | 8, 290 | 0.54 | 1.95 | .054 | .051 |

* indicates a significant effect at the 5% level.

Given the significant interaction between CONTRAST and LANGUAGE, we present the results of separate one-way ANOVAs of the factor LANGUAGE for the two contrasts. These revealed that the difference between the languages was significant only in the prosodic contrast (*p*<.001, $\eta_{p}^{2}$=.177).

**Table 3. Summary of the separate one-way ANOVAs for the segmental and prosodic contrasts.**

|  | **Sum of squares** | **Mean squares** | ***F* (4, 145)** | ***P* value** | $\boldsymbol{\eta}_{\mathbf{p}}^{\mathbf{2}}$ |
| --- | --- | --- | --- | --- | --- |
| Segmental contrast | 1.21 | 0.30 | 1.91 | .112 | .050 |
| Prosodic contrast | 12.75 | 3.19 | 7.81 * | **<.001** | .177 |

* indicates a significant effect at the 5% level.

A post-hoc Sidak test yielded two homogeneous sets, i.e. Dutch and Japanese in one set (better performance), and Persian, French and Indonesian in the other (worse performance; see Table 4).

**Table 4. Summary of a one-way ANOVA with Sidak post-hoc analysis for the prosodic contrast.**

| Language (I) | Language (J) | Mean Difference (I-J) | P-value | 95%CI |
| --- | --- | --- | --- | --- |
| Persian | Dutch | -0.50 * | **.028** | [-0.97, -0.32] |
|  | Japanese | -0.55 * | **.012** | [-1.01, -0.78] |
|  | Indonesian | 0.10 | 1.000 | [-0.37, 0.57] |
|  | French | 0.10 | 1.000 | [-0.37, 0.57] |
| Dutch | Japanese | -0.46 | 1.000 | [-0.51, 0.42] |
|  | Indonesian | 0.60 * | **.026** | [0.13, 1.07] |
|  | French | 0.60 * | **.004** | [0.13, 1.07] |
| Japanese | Indonesian | 0.64 * | **.001** | [0.17, 1.11] |
|  | French | 0.65 * | **.001** | [0.18, 1.12] |
| Indonesian | French | 0.00 | 1.000 | [-0.46, 0.47] |

* indicates a significant difference at the 5% level.

**Other interactions**

SEQUENCE LENGTH × LANGUAGE

The interaction between SEQUENCE LENGTH and LANGUAGE was significant [*F*(8, 290)=2.17, *p*=.030, $\eta_{p}^{2}$=.056] (Fig. 1). Separate one-way ANOVAs revealed that while LANGUAGE was significant at each level of SEQUENCE LENGTH ([*F*(4, 145)=4.29, *p*=.003, $\eta_{p}^{2}$=.106], [*F*(4, 145)=7.29, *p*<.001, $\eta_{p}^{2}$=.168], [*F*(4, 145)=5.31, *p*=.001, $\eta_{p}^{2}$=.128] at the 3-word, 4-word and 5-word lengths, respectively), the scores for the language groups were not uniform across the three sequence lengths (Fig. 1). Only for the 4-word sequence did we find homogeneity for the two groups.


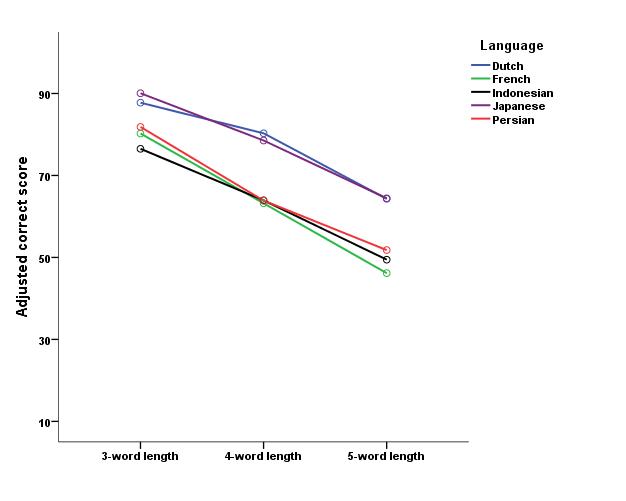


*Fig. 1- SEQUENCE LENGTH × LANGUAGE interaction*

STIMULUS TYPE × LANGUAGE

The interaction between STIMULUS TYPE and LANGUAGE was significant [*F*(4, 145)=2.59, *p*=.039, $\eta_{p}^{2}$=.067] (Fig. 2). While there were main effects of LANGUAGE for each stimulus set ([*F*(4, 145)=3.68, *p*=.007, $\eta_{p}^{2}$=.092], [*F*(4, 145)=8.53, *p*<.001, $\eta_{p}^{2}$=.191] for the Dutch Set and the Persian Set, respectively), the difference between the languages is somewhat larger for the Persian stimuli. Sidak post-hoc comparisons for the Dutch stimulus set failed to produce pairs if significantly different languages, but for the Persian stimulus set full homogeneity could be established again.

**
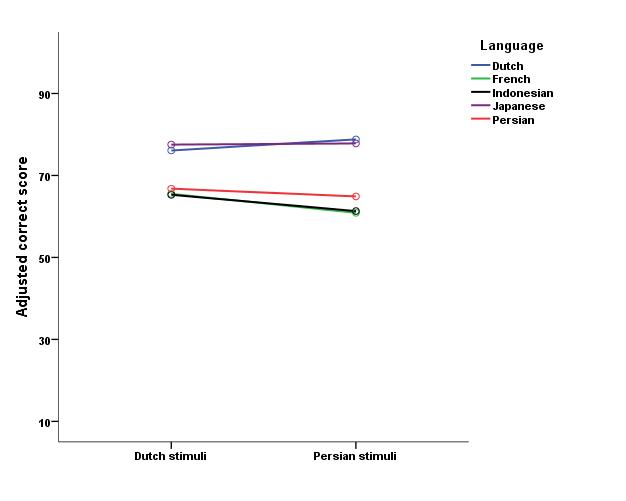
**

*Fig. 2- STIMULUS TYPE × LANGUAGE interaction*

STIMULUS TYPE × CONTRAST

The interaction between STIMULUS TYPE and CONTRAST was significant [*F*(1, 145)=5.34, *p*=.022, $\eta_{p}^{2}$=.036] (Fig. 3). This reflects the fact that while there is an effect of CONTRAST for both sets of stimuli, this effect is somewhat larger for the Persian Set.


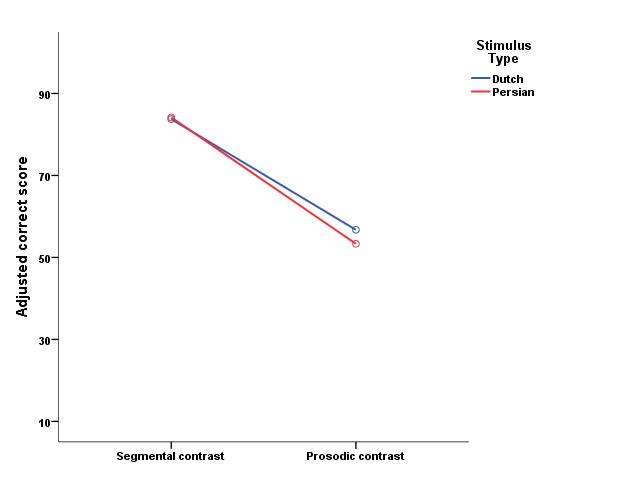


*Fig. 3- STIMULUS TYPE × CONTRAST interaction*
